# História da Memória das Coleções Científicas do Instituto Oswaldo Cruz da Fiocruz: condições de possibilidade da escrita da história das ciências

**DOI:** 10.1590/S0104-59702024000100067

**Published:** 2024-12-16

**Authors:** Rafaella Lúcia de Azevedo Ferreira Bettamio

**Affiliations:** i Pesquisadora, Centro de Pesquisa e Editoração/Fundação Biblioteca Nacional. Rio de Janeiro – RJ – Brasil rafabettamio@gmail.com

**Keywords:** Memória, Coleções científicas, História oral, Instituto Oswaldo Cruz, Casa de Oswaldo Cruz, Memory, Scientific collections, Oral history, Instituto Oswaldo Cruz, Casa de Oswaldo Cruz

## Abstract

A partir do acervo de entrevistas de história oral da Casa de Oswaldo Cruz, Memória das Coleções Científicas do Instituto Oswaldo Cruz da Fiocruz, o artigo visa refletir sobre as condições de possibilidade da escrita da história das ciências. O objetivo do estudo é traçar a história da produção dessa memória, a partir do reuso das entrevistas do acervo e da análise de novos depoimentos de história oral realizados em 2023. Especial atenção foi dada a intenções, escolhas, lacunas, entre outras operações narrativas inseridas no processo de patrimonialização dessa memória sobre as coleções científicas do Instituto Oswaldo Cruz. A abordagem insere-se no campo de investigação sobre a gestão do patrimônio cultural, dialogando com uma vertente que investe na dimensão do conhecimento científico.

## Abstract (en)

The collection of oral history interviews from Casa de Oswaldo Cruz, Fiocruz, is the starting point for a reflection on the conditions of possibility for the writing of history of science. The study traces the history of the production of this memory by drawing on interviews from the collection and making analyses of new oral history testimonials from 2023, directing special attention to the intentions, choices, gaps, and other narrative operations involved in the patrimonialization of this memory about the scientific collections from the Instituto Oswaldo Cruz. The research is affiliated to cultural heritage management studies, in particular those studies that probe the situated nature of scientific knowledge.

O presente artigo visa contribuir com as reflexões acerca das condições de possibilidade da escrita da história das ciências por meio da análise do acervo documental de história oral Memória das Coleções Científicas do Instituto Oswaldo Cruz da Fiocruz (Memória..., 1994-2001) e do seu catálogo homônimo (Almeida et al., 2001). Os depoimentos originais foram produzidos no âmbito do projeto de pesquisa da Fundação Oswaldo Cruz (Fiocruz) “A construção das tradições científicas, os acervos de biodiversidade e a produção do conhecimento: as coleções científicas da Fundação Oswaldo Cruz”, que, por ter título extenso, ficou conhecido institucionalmente como Projeto Coleções. Durou oito anos, de 1993 a 2001, e foi fomentado como Programa de Apoio à Pesquisa Estratégica em Saúde (Papes) da Fiocruz.

A pesquisa aqui apresentada vem revelar como memórias relacionadas ao processo de organização e manutenção de coleções patrimonializadas têm potencial de esclarecer significados atribuídos à narrativa científica por meio das atividades desenvolvidas em instituições custodiadoras de acervos históricos. Parte-se da noção de “ativação de arquivos” de Eric Ketelaar (2012, p.29), que elucida o fato de as ativações acontecerem por meio de qualquer tipo de interação, intervenção, interrogação e interpretação – seja do produtor, do usuário ou do arquivista –, engendrando assim significados aos arquivos. Essa ideia torna possível dimensionar com maior clareza a abrangência de significados produzidos pelas atividades executadas por essas instituições.

Tendo em vista que tais significados constituem um universo escondido que precisa ser explorado, já que é rara a percepção desses significados, mesmo a pesquisadores que se utilizam dos acervos institucionais como fontes de informação, o presente artigo faz uma leitura a contrapelo do Projeto Coleções em busca de revelar a história da memória das coleções científicas do Instituto Oswaldo Cruz (IOC). Dentro do campo de investigação sobre a gestão do patrimônio cultural, o estudo se insere no movimento que compreende tanto a produção quanto a organização de acervos como atividades narrativas. Estas que, ao privilegiarem certos temas, norteiam pesquisas, estimulam determinadas interpretações científicas e, por meio dessa dinâmica, constroem e projetam socialmente os alicerces de uma identidade institucional.

Tal qual a instituição que as conserva, as coleções científicas do IOC existem há décadas e, sem dúvida, são valiosas para as mais variadas pesquisas do campo biomédico e da história das ciências. Na produção dessas coleções, trabalharam gerações de pesquisadores e auxiliares de pesquisa em expedições científicas que percorreram o território brasileiro do início do século XX em diante. São nove “coleções científicas antigas” credenciadas pelo IOC,^1^ que desde 1900 atuam na formação e na preservação de conjuntos de amostras biológicas vivas e não vivas.

Inserido no Projeto Coleções, o subprojeto Memória das Coleções Científicas do Instituto Oswaldo Cruz da Fiocruz resultou no conjunto de 11 depoimentos orais de trabalhadores – majoritariamente ocupantes de funções de gestão – que se dedicaram à organização e manutenção de grande parte das coleções científicas antigas do IOC. A utilização desse acervo de entrevistas no presente estudo se baseia no entendimento de que as memórias de indivíduos diretamente envolvidos com a curadoria de coleções em entidades públicas refletem aspectos socioculturais que formam esses atores, a instituição em que trabalham e o contexto histórico em que estão inseridos. Quando identificados, tais elementos revelam a historicidade das fontes e, a partir dela, como influências e decisões tomadas nos bastidores de instituições custodiadoras de acervos históricos atribuem significados aos documentos custodiados e, dessa forma, são capazes de influenciar o curso da narrativa científica.

## Por trás da memória

Tenho especial interesse em refletir sobre a memória relacionada ao trabalho em entidades públicas custodiadoras de acervos históricos. Meu objetivo principal é compreender, por meio das memórias de indivíduos que atuam nessas instituições, como atividades rotineiras atribuem significados aos documentos organizados, destacados, conservados, disponibilizados, difundidos, mas também aos esquecidos, escondidos, negligenciados e até mesmo descartados de seus armazéns, salas e estantes.

O entendimento de que as memórias dos agentes responsáveis por tais acervos têm a capacidade de desnudar escolhas de cunho técnico e organizacional e demais atividades desenvolvidas no seio dessas entidades ao longo do tempo é a substância dessa investigação. Nessa direção, dediquei um segmento da pesquisa do doutorado para investigar o lugar atribuído na Fundação Biblioteca Nacional (FBN) a uma coleção que compõe o seu acervo, mas cuja organização e cujo processo de microfilmagem foram executados pelo escritório de representação da Biblioteca do Congresso norte-americano no Rio de Janeiro, sua produtora. Tal experiência de pesquisa teve influência decisiva na minha formação e na abordagem da pesquisa que originou este artigo.

A FBN foi uma das dezenas de instituições do mundo a quem cópias microfilmadas da Coleção Brazil’s Popular Groups (BPG) foram destinadas. Na tese, posteriormente publicada em livro (Bettamio, 2021), trabalhei justamente a ideia de que, na FBN, a referida coleção sofreu diversos tipos de “ativações” ao longo do tempo. Tais ativações criaram significados, deixando marcas que diferenciavam o conjunto de cópias microfilmadas que a FBN abrigava em seus armazéns dos demais conjuntos distribuídos por outras instituições mundo afora, ainda que muitas vezes fossem materialmente idênticos. O conceito “ativação de arquivos”, formulado por Ketelaar (2001), foi então levado para o universo das coleções e bibliotecas com o objetivo de identificar “rastros” da trajetória da Coleção BPG dentro do acervo da FBN, revelando reflexos de escolhas e visões institucionais muitas vezes imperceptíveis.

A noção de ativação trazida por Ketelaar evidencia como a complexa relação entre coleção/arquivo e instituição de organização e/ou conservação, entre múltiplos avanços e recuos, determina o grau de visibilidade dos artefatos em seu acervo e, por conseguinte, seus usos. A experiência anterior de pesquisa, marcada pelas dificuldades de registros sobre a trajetória da coleção em uma de suas instituições de guarda, confere valor especial à memória dos agentes envolvidos nos processos de organização, conservação e disponibilização de fundos e coleções em entidades públicas custodiadoras de acervos históricos.

A partir disso, o presente estudo configurou-se como outro grande desafio: desvendar aspectos relacionados à influência da memória e identidade institucionais na narrativa do conjunto de depoimentos produzidos pelo Projeto Coleções da Fiocruz, considerando contextos sócio-históricos em que os processos de formação e manutenção desse acervo estiveram inseridos. Partiu-se então da leitura das entrevistas que formam o seu subprojeto Memória das Coleções Científicas do Instituto Oswaldo Cruz da Fiocruz, disponibilizadas pelo Departamento de Arquivo e Documentação (DAD) da Casa de Oswaldo Cruz (COC) no repositório digital de informações sobre o acervo arquivístico permanente da Fiocruz.

Produzidas entre 1994 e 2001, as entrevistas serviram de fonte para a presente investigação, que operou o reuso daquele acervo. O interesse pelas narrativas resultantes dos encontros entre entrevistados(as) e entrevistadores(as) não é aqui o mesmo que motivou a sua produção – registrar e consolidar uma memória das coleções científicas do IOC –, mas sim compreender como as condições de enunciação daquela produção de memória estão refletidas nas entrevistas, e vice-versa. Na mesma direção proposta por Luciana Heymann e Verena Alberti (2018, p.19), entende-se que o documento pode ser alvo de múltiplas leituras, sendo as entrevistas de história oral especialmente potentes para estudos de história da memória.

Para esse “giro reflexivo”, as autoras propõem que as entrevistas não sejam vistas “apenas como testemunhos, mas relações, intenções, condicionantes institucionais, injunções sociais etc.” (Heymann, Alberti, 2018, p.19). Diante disso, “tomar entrevistas já existentes como fontes de pesquisa, nessa perspectiva, significa inquiri-las a partir de outras motivações. Seu reuso não estaria limitado à repetição de ‘verdades’ cristalizadas, ganhando destaque o caráter histórico e contingente de sua produção” (p.19).

Nessa chave de promover novas reflexões sobre fontes de memória, Benoît de L’Estoile (2011, p.37), concentrando-se no universo das coleções de museus, conclui que “o museu dos outros pode ser também um museu de si”. Por meio das coleções históricas e etnográficas, sugere pensar sobre os objetivos e as características que nortearam os museus a colecionar e abrigar os objetos que as compõem. Expõe, assim, como certas subjetividades determinam ações do colecionador e/ou curador, e como a coleta, a seleção, a organização e a distribuição espacial dos materiais colecionados refletem importantes aspectos históricos da cultura dos espaços aos quais pertencem.

Esses autores elevam a importância do papel desempenhado pelas entidades custodiadoras de acervos históricos e por seus “técnicos”, contribuindo para sustentar a criação de registros relacionados à memória dos trabalhadores dessas instituições. Afinal, por trás das memórias de seus trabalhadores, encontra-se uma série de significados que desafia a neutralidade atribuída a essas entidades dedicadas à guarda e proteção de acervos.

Característica inerente às fontes de história oral, as entrevistas produzidas no subprojeto Memória das Coleções Científicas do Instituto Oswaldo Cruz da Fiocruz são “datadas”, ou seja, estiveram voltadas para determinado projeto de pesquisa, o Projeto Coleções, e se referem à memória das Coleções Científicas do IOC correspondente aos anos 1990 e à virada do ano 2000. Logo, para servirem como fontes históricas, fez-se necessário considerar os contextos de sua produção. Desse modo, a fim de contornar a limitação relacionada à pequena quantidade de metadados disponíveis sobre a produção do Projeto Coleções (1993-2001) e das entrevistas a partir dele originadas (1994-2001), a presente pesquisa recorreu à criação de novas fontes orais, realizando entrevistas atuais (2023) com pesquisadores da COC que estiveram diretamente envolvidos na produção dos depoimentos do Projeto Coleções, colhidos durante sua execução, entre 1994 e 2001.

É sempre válido evocar Le Goff (1996) para lembrar que essa não é “prerrogativa exclusiva” do documento de história oral, que, decerto, possui uma dimensão intrínseca de monumento, presente, porém, em qualquer documento de arquivo. O historiador francês desnaturaliza o estatuto histórico dos documentos em geral, ao categorizar que “o documento é um produto da sociedade que o fabricou segundo as relações de forças que aí detinham o poder” (Le Goff, 1996, p.470). É taxativo ao afirmar que só a análise do documento como monumento permite ao historiador usá-lo cientificamente, com pleno conhecimento de causa. Com relação à reutilização de entrevistas de história oral, Heymann e Alberti apontam caminhos que seguem na direção anunciada por Le Goff. Alertam que os próprios contextos de produção das entrevistas podem se tornar objetos de investigação e sugerem algumas questões que inspiraram o estudo apresentado:

Por meio de que procedimentos algumas narrativas se transformam em enunciados dotados de estabilidade? Qual o papel do pesquisador que trabalha com história oral na consagração dessas narrativas? Como explorar entrevistas disponíveis à consulta como documentos que enquadram memórias (e não apenas como documentos que registram enquadramentos)? São questões que podem nos ajudar a refletir sobre o patrimônio documental que produzimos e sobre nossa própria atuação nesse campo (Heymann, Alberti, 2018, p.28).

Nesse sentido, este artigo reflete sobre o papel dos organizadores e pesquisadores do subprojeto Memória das Coleções Científicas do Instituto Oswaldo Cruz da Fiocruz na consagração das narrativas enunciadas e também sobre o modo como o contexto histórico do momento das entrevistas influenciou seu resultado. Contudo, atenta-se à necessidade de não atribuir fragilidade a um dos lados da entrevista, considerando sempre que a relação entre entrevistador e entrevistado é negociada desde o princípio, conforme salientado por Janaína Amado (1997, p.154):

Em vez de apresentar-se nos termos habituais, onde existe um polo poderoso e dominante (o do historiador) e um polo fraco e submisso (o do entrevistado) – o que acaba gerando culpa no primeiro, se este é um profissional ‘ético’, dotado de senso de responsabilidade social –, o que em verdade acontece é uma relação desde o início negociada, caracterizada pelas trocas entre os objetivos do historiador (escrever a pesquisa acadêmica e, se possível, torná-la em livro) e os do informante (levar a sua experiência até outros círculos sociais, via produto final do trabalho do historiador).

## Memória em camadas

Submetido à examinação institucional, o Projeto Coleções foi contemplado com o fomento oferecido pelo Papes-Fiocruz em 1993. O projeto foi assinado pelos pesquisadores Wim Degrave, coordenador principal, químico e PhD em biologia molecular vinculado ao IOC, e a cocoordenadora Marli Brito de Albuquerque, historiadora e doutora em história contemporânea vinculada à COC. Como Wim Degrave encontrava-se lotado no Laboratório de Biologia Molecular e Diagnóstico de Doenças Infecciosas do IOC, o Projeto Coleções originalmente estava vinculado a esse laboratório.

Seu subprojeto, Memória das Coleções Científicas do Instituto Oswaldo Cruz da Fiocruz, realizado entre 1994 e 2001, produziu 11 depoimentos que originaram o catálogo homônimo de entrevistas de história oral organizado em 2001 pelas seguintes pesquisadoras doutoras da COC: as historiadoras Anna Beatriz de Sá Almeida, Laurinda Rosa Maciel e Lisabel Klein, além da bióloga e doutora em história e filosofia da ciência Magali Romero Sá. No catálogo, algumas particularidades atribuídas a cada entrevista chamam a atenção por refletirem escolhas dos organizadores e pesquisadores envolvidos no subprojeto. Após a fotografia e biografia do entrevistado, bem como o sumário da entrevista, há destaque a certas qualidades dos depoimentos, tais como a data da entrevista, a quantidade de encontros firmados com o entrevistado, a coleção científica a que sua trajetória está relacionada e os nomes dos entrevistadores, conforme resumido no Quadro 1.

**Quadro 1:** : Configuração geral dos depoimentos orais

Entrevistado	Coleção IOC	Data da entrevista	Entrevistadores
Anna Kohn Hoineff	Coleção Helmintológica	4/6/2000 e 5/7/2000	Laurinda Rosa Maciel; Magali Romero Sá; Nathacha Reis
Delir Corrêa Gomes Maués da Serra Freire	Coleção Helmintológica	24/11/2000	Anna Beatriz de Sá Almeida; Magali Romero Sá
Dely Noronha de Bragança Magalhães Pinto	Coleção Helmintológica	1/2/2000	Anna Beatriz de Sá Almeida; Laurinda Rosa Maciel; Nathacha Reis
Dyrce Lacombe de Almeida	Coleção Entomológica	2/7/1999 e 11/8/1999	Anna Beatriz de Sá Almeida; Magali Romero Sá; Renata Fernandes Marques
Herman Lent	Coleção Entomológica	12/9/1994 a 23/11/1994	Flávio Edler; Wanda Hamilton
Itália Guarany Angiola Kerr	Coleção de Febre Amarela	1/4/1998 e 29/4/1998	Anna Beatriz de Sá Almeida; Magali Romero Sá
José Jurberg	Coleção Entomológica	25/11/1999 e 31/11/1999	Anna Beatriz de Sá Almeida; Magali Romero Sá
Maria Inez de Moura Sarquis	Coleção de Cultura de Fungos	8/2/2001	Anna Beatriz de Sá Almeida; Magali Romero Sá
Orlando Vicente Ferreira	Coleção Entomológica	21/02/2001	Anna Beatriz de Sá Almeida; Magali Romero Sá
Sebastião José de Oliveira	Coleção Entomológica	8/11/2000	Anna Beatriz de Sá Almeida; Magali Romero Sá
Wladimir Lobato Paraense	Coleção Malacológica	3/6/1998 a 1/07/1998	Anna Beatriz de Sá Almeida; Magali Romero Sá
**11 entrevistados**	**5 coleções**	**1994-2001**	**7 entrevistadores**

Fonte: Almeida et al., 2001.

Apesar de serem nove coleções científicas antigas credenciadas pelo IOC, o quadro relaciona apenas cinco delas aos 11 entrevistados, sem mencionar as demais coleções que integravam o patrimônio institucional naquele momento. Já no texto do Projeto Coleções, ou Projeto Papes-Fiocruz (1993), sete coleções científicas são referenciadas, além de outras coleções e conjuntos tipológicos mais específicos.^2^

Na sequência, o texto apresenta os objetivos do projeto: em primeiro lugar, definir o conteúdo de cada coleção quanto à sua base documental, relacionar os dados obtidos com os registros históricos, para, em seguida, partir para a divulgação das informações obtidas e, finalmente, indicar caminhos para abordagens mais profundas. Não há esclarecimentos sobre o que motivou a seleção das coleções científicas que seriam submetidas a esse aprofundamento. No entanto, na abordagem utilizada nas entrevistas, encontram-se algumas pistas.

Os coordenadores do projeto sugerem que, naquele momento, o potencial dos bancos de referência e coleções estava gravemente subutilizado. Frisam, por outro lado, a existência de exceções a esse panorama, pois já haviam sido informatizados o banco de isolados *bacillus* do Departamento de Bacteriologia, o Centro de Referência de Leishmania e as coleções do Instituto Nacional de Controle de Qualidade em Saúde (INCQS). Segundo eles, até aquele momento, “nenhuma outra coleção ou banco de amostras da Fiocruz foi devidamente catalogado e anotado de forma sistemática, e muito menos foram estes dados colocados à disposição da comunidade científica” (Degrave, Albuquerque, 1993, p.6.3). Desse modo, os coordenadores do projeto constroem o argumento sobre a urgência do trabalho proposto e apontam para o motivo de as coleções Leishmania, Bacillus e de Culturas do INCQS não estarem no rol de prioridades.

Ao relatarem possíveis limitações do projeto, os coordenadores admitem que nem todos os enfoques propostos poderão ser completados em dois anos, tempo previsto para o financiamento. De fato, a extensão do trabalho acaba se estabelecendo, já que este é finalizado apenas em 2001, com a publicação do catálogo e a disponibilização das entrevistas. Afirmam ainda que, diante das limitações previstas, “algumas coleções com conteúdo e processo históricos mais interessantes serão escolhidas para estudos imediatos mais profundos”, e acrescentam que “a escolha destas coleções será feita durante o decorrer do projeto” (Degrave, Albuquerque, 1993, p.6.6).

Ainda que no projeto não se tivesse definido quais seriam as coleções beneficiadas, as entrevistas de sua segunda fase deixam claro que cinco coleções científicas do IOC foram “escolhidas” para o aprofundamento vislumbrado em 1993. No entanto, a representatividade das coleções contempladas pelas entrevistas sugere que o aprofundamento que as motivava não ocorreu de forma equânime.

A Coleção Entomológica está representada por cinco entrevistados, responsáveis em algum momento pelo conjunto colecionado. Em seguida, três pesquisadoras entrevistadas configuram a Coleção Helmintológica, todas curadoras do conjunto no passado ou no momento das entrevistas. Já as três coleções restantes – Febre Amarela, Malacológica e Culturas de Fungos – estão simbolizadas por um entrevistado cada, todos curadores no momento das entrevistas.

Nas primeiras páginas do catálogo, as organizadoras, Magali Romero Sá e Lisabel Klein (2001), apresentam o projeto original Papes (1993-2001), destacando as atribuições de suas diferentes fases e, ao mesmo tempo, ressaltando o seu caráter multidisciplinar, envolvendo especialmente as ciências humanas e as biológicas. De fato, em 1993, essa multidisciplinaridade era uma característica ainda inovadora para um projeto interno da Fiocruz. A instituição, àquela altura quase centenária, tinha no IOC, com a pesquisa concentrada nas ciências biomédicas, o seu núcleo de criação. Já a COC, unidade inaugurada em 1986, era ainda uma novidade em pleno estabelecimento, voltada para a valorização da memória da Fiocruz e para as atividades de pesquisa, ensino, documentação e divulgação da história da saúde pública e das ciências biomédicas no Brasil:

Com identidade multidisciplinar, unindo as áreas das ciências humanas e biológicas, este trabalho contou com uma equipe formada por historiadores, arquivistas e biólogos curadores de coleções e procedeu ao levantamento das coleções e organização da documentação histórica gerada ao longo do processo de sua constituição. Adicionalmente, constituiu acervo de depoimentos orais, através de entrevistas com curadores, ex-curadores, pesquisadores e técnicos que trabalharam ou trabalham junto às coleções.

Para garantir a abrangência proposta, o projeto foi subdividido em duas fases distintas: a primeira, que correspondeu ao período de 1994-1996, constitui basicamente na implementação de um programa de informatização das coleções e no levantamento preliminar e do material documental existente. Na fase seguinte, desenvolvida entre 1997 e 2001, deu-se continuidade à informatização, procedeu-se a identificação e organização da documentação sob a guarda do Departamento de Arquivo e Documentação da Casa de Oswaldo Cruz, além de constituir um acervo de depoimentos orais (Sá, Klein, 2001, p.10).

As demais organizadoras do catálogo, Anna Beatriz de Sá Almeida e Laurinda Rosa Maciel, apresentam os principais eixos e as questões que o conjunto de depoimentos agrega ao projeto. Relacionam também as cinco coleções científicas do IOC que ali estão representadas pelos trabalhadores entrevistados. A apresentação de Almeida e Maciel não explicita o motivo da escolha de determinadas coleções em detrimento de outras e, desse modo, contrasta com as últimas páginas do catálogo, nas quais há os créditos referentes à equipe de pesquisa das duas fases do projeto e a lista de “Curadores das Coleções do IOC/Fiocruz”, que enumera as nove coleções científicas existentes no IOC (Almeida et al., 2001, p.68-69).

A princípio, a hipótese levantada é que a lacuna de informação sobre a prioridade dada a certas coleções e entrevistados seria uma forma de omissão narrativa que refletiria motivações ocultas e, ao mesmo tempo, engendraria significados sobre o acervo de entrevistas e das coleções, uma vez que atribui importância e projeção institucionais a certas coleções e sujeitos em detrimento de outros. No entanto, por meio da combinação de informações colhidas de outras fontes, foi possível perceber que determinadas escolhas podem ter sido pautadas por questões práticas referentes a diferentes graus de conservação, processamento técnico e informacional das coleções científicas, além do tamanho de cada conjunto. Por exemplo, quatro coleções não contempladas com entrevistas no subprojeto se referem justamente aos conjuntos enunciados no projeto original (1993) como os únicos com informatização e sistematização de dados disponíveis na ocasião.^3^

Nesse sentido, o silêncio no catálogo de entrevistas (Almeida et al., 2001) sobre a escolha das coleções e dos entrevistados parece traduzir mais a problemática da produção geral de história oral da época do que um silenciamento consciente dos produtores. Nos programas e acervos de história oral do final dos anos 1990 e início dos anos 2000, ainda não havia uma definição sobre a importância de se esclarecer as condições que promoveram o projeto e sustentaram a realização de suas entrevistas. Vale destacar que, mesmo em tempos recentes, a divulgação desses metadados ainda é precária. Sobre esse ponto, Heymann e Alberti constatam que a consulta a esses acervos é, em geral, inferior às expectativas criadas por suas instituições promotoras e que, embora o reuso das entrevistas aconteça, é inversamente proporcional ao investimento direcionado à sua preservação e disponibilização. Desse modo, sinalizam para a necessidade de que, na atualidade, as instituições considerem disponibilizar os contextos de produção das entrevistas de história oral, destacando que os próprios contextos podem se tornar objetos de investigação e incentivar, assim, o reuso dessas fontes (Heymann, Alberti, 2018, p.27-28).

No Projeto Coleções, a primeira fase foi dedicada à informatização das coleções e ao levantamento da documentação existente, e a segunda, à continuidade e ao aprofundamento desse trabalho. Diante da limitação de recursos orçamentários e humanos, faz sentido que a preferência por colher mais informações históricas fosse direcionada àquelas coleções científicas que ainda não estivessem minimamente conservadas, catalogadas e informatizadas. Contudo, por que não explicitar o motivo dessa escolha no catálogo das entrevistas? Seria explicada somente pelo tipo de história oral efetuado na época pela instituição, no qual não havia ainda se estabelecido a consciência da importância dos metadados da pesquisa? Ou também teria relação com a memória coletiva e a cultura institucionais consolidadas de tal forma que a explicação parecesse a seus produtores um dado evidente?

A busca por esses motivos impulsionou a produção de novas entrevistas que trouxessem mais elementos sobre o processo de construção da memória das coleções científicas do IOC desenvolvido no contexto do Projeto Coleções. Assim, tornou-se mais factível o exercício de colocar essa memória em perspectiva, desvelando algumas de suas camadas para compreender a sua história. Além de fonte de informação, a história oral foi também um recurso metodológico essencial ao aprofundamento da investigação. Afinal, para a compreensão desse processo, foi fundamental entrevistar pesquisadores(as) da COC que atuaram também como entrevistadores(as) no projeto entre 1994 e 2001.

## Entrevistadores no passado, entrevistados do presente

Para se traçar a história da memória das coleções científicas do IOC, compreender o fenômeno da memória é essencial. Ter em mente que os relatos expõem processos de sistematização da memória, constituída pela negociação entre dimensões coletivas e individuais (Pollak, 1989, 1992) que se apresentam mediante construção discursiva orientada pelo presente (Sarlo, 2007; Delgado, 2003) é decisivo tanto para a releitura dos depoimentos quanto para as entrevistas realizadas no presente.

Fernando Catroga, ao relembrar a afirmação de Umberto Eco de que o homem é um animal histórico, destaca que indivíduos necessitam da temporalidade, sendo essa uma das funcionalidades da assimilação da memória. No entanto, a memória não é “um armazém inerte, onde, por ocasional e arbitrária acumulação, se recolhem os acontecimentos vividos por cada indivíduo”; ao contrário, “ela é retenção afetiva e quente dos traços inscritos na tensão tridimensional do tempo – passado-presente-futuro – que permanentemente a tece” (Catroga, 2015, p.16-17). A partir da perspectiva do “trabalho de memória” de Ricoeur (2007, p.99-105), Catroga (2015, p.53-54) reflete então que a memória e a história estão sujeitas a interferências da sociedade, do tempo, de subjetividades, técnicas, entre outros fatores, relativizando, assim, a oposição sistemática entre história e memória.

A partir da consciência das interferências e das marcas dessa tensão tridimensional do tempo, as quais as memórias trazidas nos depoimentos e também a produção da história estão sujeitas, é que se compreende a historicidade e, desse modo, a singularidade temporal tanto das entrevistas analisadas quanto da própria abordagem histórica travada por essa pesquisa. Ainda que de maneiras distintas, as memórias e a história refletem escolhas pautadas no presente por sujeitos, instituições, contextos sociopolíticos, relações interpessoais, entre outras influências contextuais a que se vinculam. Assumir que essa perspectiva inclui o próprio caminho aqui traçado é indispensável para compreender, por exemplo, a escolha dos cinco pesquisadores da COC entrevistados em 2023 para esta análise.

A pesquisa aqui descrita é decorrente do estágio pós-doutoral realizado no Departamento de Arquivo e Documentação da COC, unidade da Fiocruz, cujo corpo funcional é composto majoritariamente por historiadores e, em menor medida, por sociólogos, museólogos e arquivistas. Além propriamente do tema da pesquisa, há uma proximidade geográfica e acadêmica entre mim, historiadora, e meus cinco entrevistados: quatro historiadores e uma socióloga, que participaram de uma ou duas fases do Projeto Coleções e cumpriram em algum momento o papel de entrevistadores no seu subprojeto de história oral.

Os cinco pesquisadores da COC entrevistados foram: Flávio Edler (historiador, pesquisador da primeira fase), Magali Romero Sá (bióloga e historiadora, coordenadora e entrevistadora da segunda fase), Laurinda Rosa Maciel (historiadora, pesquisadora das duas fases e entrevistadora na segunda fase), Anna Beatriz de Sá Almeida (historiadora, pesquisadora e entrevistadora da segunda fase) e Wanda Hamilton (socióloga, entrevistadora convidada na primeira fase). Hamilton foi a única que não integrou o Projeto Coleções propriamente. Sua atuação limitou-se a de entrevistadora convidada na primeira entrevista. Ao lado de Flávio Edler, este sim pesquisador integrante do projeto durante a primeira fase, Wanda Hamilton entrevistou, em 1994, o parasitólogo Herman Lent, um dos grandes responsáveis pela Coleção Entomológica do IOC por décadas.

Com relação ao formato geral dos depoimentos produzidos na segunda fase do projeto, entre 1997 e 2001, há muitas especificidades na entrevista de Herman Lent. Ela ocorreu entre setembro e novembro de 1994, num momento em que o Projeto Coleções ainda estava em sua primeira fase. Conforme já visto, as entrevistas não haviam sido especificadas no texto do Projeto Papes (1993), pois seriam definidas mais adiante, em função de uma abordagem aprofundada das coleções já trabalhadas na primeira etapa do projeto.

O caráter antecipado da entrevista de Herman Lent e o fato de Wanda Hamilton estar entre seus entrevistadores traduzem o destaque dado a esse depoimento pelo projeto naquele momento. Wanda havia atuado recentemente como uma das organizadoras de outro trabalho, o Memória de Manguinhos, um dos projetos de história oral fundadores da COC, que havia sido inaugurada em 1986. O catálogo de entrevistas originado por aquele projeto foi publicado em 1991, apresentando acervo extenso de depoimentos de personalidades ligadas à história do IOC (Britto, Hamilton, 1991). Realizadas entre 1986 e 1989, as entrevistas trataram de temas relacionados “ao período compreendido entre a década de 1930 e o Massacre de Manguinhos, em 1970” (Gadelha, 1991, p.VIII).

*O Massacre de Manguinhos* é o título do livro de Herman Lent, de 1978, no qual ele narra e analisa os fatos relacionados ao episódio de 1970, quando, durante a ditadura civil-militar (1964-1985), um grupo de pesquisadores cientistas do IOC foi compulsoriamente aposentado e teve os direitos políticos suspensos por dez anos, entre eles, o próprio Lent.^4^ Em 1986, ocorreu o retorno aos quadros do IOC dos cientistas afastados. Lent, contudo, não aceitou retomar o seu antigo posto, permanecendo na Universidade Santa Úrsula, que o havia acolhido durante os anos de cassação. Participou, entretanto, do Conselho Técnico-Científico da instituição de 1985 a 1988, além de frequentar laboratórios do IOC até pouco antes de falecer, em 2004.

No pós-ditadura, o início da história da COC (1986) se entrelaça com o registro da memória dos pesquisadores afastados que haviam retornado. O projeto Memória de Manguinhos representava um dos três núcleos de trabalho desenvolvidos na COC em seus primeiros anos de existência, conforme esclarece o pesquisador Flávio Edler, que ali trabalha desde 1987:

Havia outro projeto que era o Memória de Manguinhos … Tinha o grupo que era mais de arquivo e de documentação, outro grupo, que era voltado à pesquisa e que fazia história oral do Inamps [Instituto Nacional de Assistência Médica da Previdência], e o outro que era esse grupo que ficava um pouco correndo atrás da história, principalmente da história oral do Instituto Oswaldo Cruz. Também era o período do retorno daqueles cientistas que foram cassados, ficou conhecido, criaram esse nome… Massacre de Manguinhos ... De fato, foi o que o governo militar fez em muitas áreas, acabou com muitas áreas de conhecimento, fez várias intervenções em universidades públicas, federais e estaduais, e tinha feito aqui, em Manguinhos, expulsado vários cientistas (Edler, 18 jan. 2023).

A formação da COC foi uma das reações ao momento traumático vivido pela instituição, era parte de sua redemocratização. Assim como ocorria em outros segmentos sociais do país, havia na Fiocruz um movimento de consolidação da memória e da verdade voltada para a ciência e a saúde, enquanto direitos reivindicados, conforme assinala o jurista José Carlos Moreira da Silva Filho:

Remonta à segunda metade do século XX a crescente afirmação de um Direito à Memória e à Verdade, configurando-se claramente como um direito transindividual, que ultrapassa a formulação por meio dos atores políticos tradicionais como partidos e sindicatos, alcançando os mais diversos grupos da sociedade civil e experimentando as mais diversas formas de reivindicação e concretização (citado em Gallo, 2010, p.137).

As memórias do IOC e do Massacre de Manguinhos estão na origem da história da COC na Fiocruz. Foi devido ao seu trabalho no projeto Memória de Manguinhos que Wanda Hamilton se tornou especialista na temática do Massacre, pois havia se envolvido com as entrevistas de todos os atingidos no episódio, com exceção de Herman, que, segundo ela,

não quis dar entrevista na época, com o argumento de que ele já tinha escrito o livro e que ele estava chateado… ... estava chateado porque a coleção, da qual foi curador, a Entomológica, estava de mudança de local, porque foi muito maltratada na época da Ditadura Militar...

... não participei nem da elaboração do Projeto Coleções nem do processo de pesquisa, eu não fazia parte do projeto. Mas, como eu quis entrevistar o Herman, eu conhecia a história do Massacre, eu tinha publicado artigo sobre esse período, então eu tinha ideia do que tinha acontecido com as coleções, com as publicações, com os pesquisadores… (Hamilton, 16 mar. 2023).

A entrevista de Lent teve a maior duração do conjunto, estendendo-se por sete encontros em sala da Universidade Santa Úrsula – que totalizam 8h46 de gravação. Essa, bem como as outras excepcionalidades apresentadas, reflete a proeminência conferida à memória de Lent pelo Projeto Coleções. A COC “devia” o registro institucional dessas memórias ao projeto Memória de Manguinhos e, sobretudo, à própria Fiocruz. Em interface com o conceito de Pollak, o destaque dado a Lent no projeto provém de elementos constitutivos de uma narrativa que traduz e, ao mesmo tempo, investe num enquadramento institucional de memória:

O trabalho de enquadramento da memória pode ser analisado em termos de investimento. Eu poderia dizer que, em certo sentido, uma história social da história seria a análise desse trabalho de enquadramento da memória. Tal análise pode ser feita em organizações políticas, sindicais, na Igreja, enfim, em tudo aquilo que leva os grupos a solidificarem o social. ... Além do trabalho de enquadramento da memória, há também o trabalho da própria memória em si. Ou seja: cada vez que uma memória está relativamente constituída, ela efetua um trabalho de manutenção, de coerência, de unidade, de continuidade, da organização (Pollak, 1992, p.206).

No Projeto Coleções, o trabalho de enquadramento e manutenção da memória institucional também é percebido pela quantidade de entrevistas relacionadas à Coleção Entomológica. Na entrevista de Lent e dos quatro outros pesquisadores a ela elencados, evidencia-se um discurso comum e veemente relativo à proteção dos materiais colecionados. Essa conformidade narrativa transparece uma identidade do grupo da entomologia do IOC representado no projeto e, ao mesmo tempo, na percepção, por parte dos entrevistados, assim como dos entrevistadores, de que as entrevistas são uma forma de agir em prol da conservação da coleção no tempo. A narrativa das entrevistas se desenrola em direção à valorização das coleções científicas, especialmente a Entomológica, de fato, uma das maiores e mais antigas coleções do IOC, a maior da América Latina – com cerca de cinco milhões de espécimes da fauna brasileira e de outras regiões do mundo, apresentando quase todas as ordens de insetos conhecidas –, além de ter sido uma das mais ameaçadas durante a ditadura civil-militar:

O ‘massacre’ não se limitou apenas à expulsão de renomados cientistas da instituição; toda a estrutura física que havia foi integralmente desmantelada, e os armários contendo material científico foram transportados em condições inadequadas para o porão do antigo prédio do Hospital Evandro Chagas, no *campus* de Manguinhos, ocasionando perdas e danos irreparáveis a inúmeros exemplares da coleção. Naquele momento, algumas partes do acervo foram enviadas para outras instituições com o intuito de protegê-las e também para darem suporte a projetos de pesquisa em andamento.

… Em 1977, uma grande parte do acervo que havia sido depositado no Hospital Evandro Chagas retornou ao segundo andar do Pavilhão Mourisco, graças ao empenho de José Jurberg e ao apoio do técnico de laboratório Orlando Vicente Ferreira. Este tinha sido assistente do mais eminente dos entomólogos brasileiros, Ângelo Moreira da Costa Lima, que iniciou sua carreira em Manguinhos em 1913 (Costa et al., 2008, p.402).

Nessa linha, o pesquisador José Jurberg, atualmente chefe do Departamento de Entomologia do IOC, em sua entrevista para o projeto, colocou-se de forma enfática em favor da conservação da Coleção Entomológica. Jurberg foi responsável pela Seção de Entomologia de 1976 a 1986, quando o pesquisador cassado Sebastião José de Oliveira assumiu a curadoria, após a sua reintegração. Jurberg (1999, p.66), em seu depoimento, destacava a situação da coleção no período da ditadura, quando Rocha Lagoa foi ministro da Saúde:

Em 1970, quando o Rocha Lagoa cassou todos os pesquisadores e resolveu transferir a coleção do Castelo, do segundo andar, para o hospital no térreo … . Então, a coleção ocupou 80% daquele hospital. Foi feita de uma maneira atabalhoada e se perdeu muito material. E o Orlando é o responsável pela existência ainda da coleção, porque senão tinha sido muito mais destruída.

É interessante notar também que a ênfase atribuída pelo Projeto Coleções às coleções Entomológica e Helmintológica reflete fatores institucionais menos visíveis. Vale lembrar que o projeto foi fruto de uma primeira parceria entre a COC, unidade organizacional criada alguns anos antes, e o IOC, unidade que deu origem à Fiocruz. Essa parceria intrainstitucional era exigida para o fomento Papes, mas não neutralizava eventuais tensões e disputas oriundas de diferenças de pensamento entre as áreas das unidades. De certo modo, alguns limites narrativos foram gerados por essa necessidade de conciliação entre as partes e influenciaram o desenho do projeto, como, por exemplo, a escolha de quais seriam as coleções contempladas e os entrevistados que as representariam.

Segundo a pesquisadora e, na ocasião, entrevistadora Anna Beatriz de Sá Almeida, os depoimentos do projeto foram estabelecidos, de forma geral, em função do critério das coleções mais procuradas por especialistas e, em alguma medida, para suprir uma necessidade de reconhecimento institucional demandado pelos curadores das coleções científicas. A partir dessas diretrizes gerais, os pesquisadores da COC estruturaram o quadro de entrevistas durante o andamento do projeto, tendo em vista os interesses dos especialistas que procuravam as coleções para pesquisa, dos seus curadores e também da própria COC. Sobre esse processo, vale a pena destacar alguns trechos da entrevista de Anna Beatriz:

Então, o que era notório na Fiocruz? Quais eram as coleções mais procuradas pelos especialistas do Brasil e de fora pra se fazer consultas e trocas de material? Empréstimos de material? A Entomológica todo mundo sabia que era muito importante, que tinha começado com o Oswaldo Cruz … era uma coleção que a gente queria pegar duas gerações, pegar pessoal do Sebastião e o José Jurberg. No meio disso, quando a gente fez a entrevista com o Zé Jurberg, ou com o Sebastião, ambos falaram que não podíamos deixar de entrevistar o professor do desenho, Orlando Vicente Ferreira...

… a Casa, quando foi criada, eram dois projetos-cabeça: o primeiro era o Memória de Manguinhos, dos cassados. Então a Malacologia, que a gente também dedicou muita energia, e a Helmintologia, foram escolhidas em função do que aconteceu com as pessoas que faziam parte delas. Então, a Malacologia já tinha uma entrevista enorme dentro do Memória de Manguinhos, que talvez não fosse necessário fazer novamente, mas a gente leu a entrevista e viu que pouco foi perguntado sobre o processo de constituição e guarda, da importância da coleção. Por isso a gente voltou pra entrevistar o Wladimir Lobato Paraense.

… todos os curadores reivindicavam: ‘Nós, curadores, temos que ter atenção, verba’.

... na verdade, a gente pegou a Febre Amarela porque ela era completamente ignorada e a gente queria dar a ela o espaço de reconhecimento em função da doença.

… A Coleção Helmintológica tinha um lugar reconhecido por todos os outros curadores, que era até estranho, mas as pessoas falavam: ‘Nossa, caramba, as meninas são fundamentais, porque ele [Lauro Travassos] formou as meninas ... Delir, Dely e Anna Kohn’ (Almeida, 30 mar. 2023).

É importante destacar também que nos depoimentos dos pesquisadores da COC, colhidos em 2023, transparece o sentimento relacionado a certa incompreensão, e até mesmo resistência, dos pesquisadores do IOC com relação ao trabalho desenvolvido por eles e ao que este poderia agregar à Fiocruz. Além disso, havia ainda a percepção de que, para o IOC, “a Casa de Oswaldo Cruz era mais uma unidade pra dividir o bolo de orçamento” (Almeida, 30 mar. 2023). Esse desconforto, sentido na época pelos pesquisadores da COC envolvidos no projeto, teria, segundo eles, impulsionado a entrada de Magali Romero Sá como coordenadora do projeto na segunda fase, em representação à COC:

A primeira vez que eu vim trabalhar aqui na instituição foi como bolsista. ... era estudante de graduação e vim fazer estágio aqui. … porque sou bióloga de formação, então eu vim trabalhar na biologia na década de 1970 ... fiquei um ano nesse laboratório ... pedi transferência e fui trabalhar com coleções científicas. Aí, minha trajetória com coleções se inicia. Fiquei um ano trabalhando com o Orlando [Guerra] com a coleção científica do doutor Hugo de Souza Lopes [Coleção Entomológica], ajudando a organizar, entendendo o que era aquilo.

Magali ajudava a gente a conversar, era uma pessoa importante nas entrevistas. Porque existe todo um saber, você não pode, quando vai fazer uma entrevista, não saber absolutamente nada do que o cara está falando. Porque não flui. Ele fala uma coisa e você ‘ahan’, fica com cara de paisagem, não sabe muito bem direito o que é, ele já vai falar: ‘Essa pessoa não está entendendo nada do que eu estou falando, estou falando para as paredes’. Então, a gente, claro, que é da área de humanas, faz um esforço de compreensão, mas é muito melhor ter uma pessoa que tem a formação da área. Ela é bióloga de formação (Maciel, historiadora, COC-Fiocruz, 2023).

Por meio dos depoimentos colhidos em 2023 e da participação de Magali Romero Sá como entrevistadora de nove das 11 entrevistas realizadas na segunda fase do Projeto Coleções, percebe-se que, de fato, a sua entrada como coordenadora do projeto foi importante, possibilitando maior articulação e troca entre as duas unidades da Fiocruz. Estimulou, inclusive, os pesquisadores da COC a se envolverem e se aprofundarem em temáticas relacionadas às coleções científicas do IOC, atribuindo novos significados àquele material.

No trecho a seguir, extraído do depoimento do pesquisador Flávio Edler, tem-se um bom exemplo de desdobramentos promovidos pelas articulações do Projeto Coleções. Foi esse envolvimento que despertou em Edler o interesse pela história da helmintologia no Brasil, tema que veio a desenvolver em seu doutorado e que resultou em importantes desdobramentos científicos.^5^ Para isso, foi essencial a aproximação com os materiais da Coleção Helmintológica, proporcionada pelo projeto e impulsionada por Magali:

Magali, já com articulação dentro do IOC, também entrou no projeto … já estava na COC, porque ela havia se formado em história da ciência na Inglaterra, então ela trouxe isso ... ela foi uma das pessoas que me empolgou. … desse projeto, surgiu meu tema de doutorado. Interessante porque aí eu me articulei ao grupo da helmintologia e resolvi fazer, construir uma história da helmintologia no IOC. ... Mas você vê, que o estudo veio a partir disso, é um desdobramento da história desse projeto (Edler, 18 jan. 2023).

Magali Romero Sá começou então a coordenar o núcleo de trabalho da COC na segunda fase do projeto. A presença de Magali em nove das 11 entrevistas ocorre quando ela já exercia, portanto, essa coordenação. Sua função central pode ser explicada pelo fato de sua formação, seu conhecimento e sua entrada junto aos pesquisadores do IOC serem, à época, qualidades fundamentais para ultrapassar as barreiras já encontradas por historiadores em visitas aos laboratórios, em negociações com pesquisadores, no levantamento de documentação histórica, na seleção e no acordo de transferência desses conjuntos ao Departamento de Arquivo e Documentação da COC:

Não andava, o projeto não andava. Eles não conseguiam entrar nos laboratórios, eles marcavam e as pessoas não aceitavam. As pessoas não deixavam, isso desestimula o próprio historiador. ... E, com esse meu perfil, além de ser bióloga, de ter um contato direto com esses pesquisadores, fui, na realidade, bolsista deles, do IOC, quando eu era jovenzinha e não existia a COC.

... Então, eu tive essa entrada muito forte dentro do projeto. Por isso eu fui chamada quase que assim ‘pelo amor de Deus, assume isso porque é importante’ (Sá, 25 jan. 2023).

A parceria de Magali com Anna Beatriz na maioria das entrevistas é decorrente da combinação entre a formação profissional e acadêmica de ambas. Anna Beatriz tem formação em história e já tinha bastante experiência com entrevistas de história oral: “Eu fiz doutorado em história das ciências no exterior, mas eu não sou uma historiadora de formação. Então, ficou trabalhando comigo a Bela [Anna Beatriz de Sá Almeida] na história oral, para me ajudar, obviamente, sobre o que é a metodologia da história oral” (Sá, 25 jan. 2023).

Anna Beatriz teve também significativa atuação como diretora do sindicato da Fiocruz, a Associação dos Servidores da Fundação Oswaldo Cruz (Asfoc), entre 1994 e 1997, período referente à primeira fase do Projeto Coleções. Essa posição a fez circular por outras unidades e se tornar conhecida entre colegas do IOC. Após sua saída do sindicato, de volta à função de pesquisadora da COC, Anna ingressa na segunda fase do projeto. A experiência sindical de Anna também auxiliou na abordagem dos pesquisadores do IOC e no desenvolvimento das entrevistas:

Eu tinha experiência com história oral, a fala das pessoas que tinham trabalhado comigo na história oral era que eu me dedicava de corpo e alma, então, independente da questão, eu ia estudar. … sabiam que iam colocar uma pessoa que ia conseguir entender do assunto pra fazer as entrevistas. A grande surpresa foi perceberem que eu já tinha entrado em alguns lugares, que eu já era recebida por meu apelido por algumas pessoas. Eu tinha um reconhecimento, porque eu tinha ido ao IOC pra entender quais eram os problemas das condições de trabalho, porque, dentro do sindicato, a gente também fazia isso. A gente ia, mostrava aqueles corredores abarrotados de geladeiras, onde a pessoa tinha que passar de lado, e, se acontecesse algum curto-circuito, se perderiam todas as amostras. Então, assim, eu já era conhecida de muitas pessoas. Na verdade, a Casa tomou conhecimento disso durante o processo. Foi uma prova, né?! Eu fui colocada à prova (Almeida, 30 mar. 2023).

Em 2023, quando concederam as entrevistas para a presente pesquisa, os pesquisadores da COC vivenciavam realidades – profissional, institucional, pessoal ou mesmo nacional – diferentes do período em que contribuíram com o Projeto Coleções (entre 1993 e 2001). No âmbito profissional, esses pesquisadores atualmente têm reconhecimento, dentro e fora da Fiocruz. Institucionalmente, a COC é uma unidade técnico-científica da Fiocruz com sólido prestígio em suas atividades de pesquisa, ensino, documentação e divulgação da história da saúde pública e das ciências biomédicas no Brasil. Nesse sentido, as memórias relacionadas ao Projeto Coleções, colhidas no tempo presente, permitem perceber que houve um processo percorrido pela COC e por seus pesquisadores até que eles se consolidassem no patamar institucional e científico atual e que o Projeto Coleções teve papel significativo nessa trajetória.

Revelando e contribuindo para amenizar as resistências internas enfrentadas pela COC, recém-criada, e por seus pesquisadores, recém-contratados, o Projeto Coleções foi para os profissionais da unidade um marco nesse trajeto. É interessante notar que, quando perguntados sobre a importância do projeto para a COC, todos os pesquisadores ouvidos refletem e chegam à mesma conclusão: o Projeto Coleções foi uma porta de entrada de reconhecimento institucional de que a COC precisava. Permitiu que outras unidades, em especial o IOC, conhecessem o trabalho dos historiadores, dando oportunidade para que começassem a percebê-los como aliados para preservar e notabilizar os materiais científicos produzidos e colecionados nas outras unidades, bem como as suas memórias. No trecho a seguir, Laurinda Maciel evidencia a projeção institucional positiva conferida pelo projeto:

O projeto foi bastante importante, porque angariou grana, a gente teve muita verba. A gente pôde entrar nos laboratórios do IOC, onde, inegavelmente, a gente tinha, assim, alguma resistência, sensibilizá-los para a importância do trabalho de preservação documental e tudo mais.

... A Marli, que foi a coordenadora na primeira fase, vivia dizendo pra gente: ‘Nós não podemos fazer feio, temos que fazer bonito o nosso papel!’. Então tinha essa preocupação, de a gente fazer uma coisa bacana, que fizéssemos bonito e ficássemos bem diante dos olhos… porque, obviamente, que um projeto desses, as pessoas falam dele. Vai, por exemplo, para um CD [Conselho Deliberativo] da instituição, da Casa, da Fiocruz, e você ganhando, vamos dizer assim, a simpatia, no sentido de um diretor do IOC ou de outros laboratórios entenderem a importância desse trabalho, entenderem porque é bacana você preservar aquilo, quais frutos pode ter daquilo, que trabalhos pode fazer a partir daquelas fontes… então, acho que tudo isso, quando chega num CD da Fiocruz, fala também sobre isso... Consolida melhor uma imagem institucional.

... Foi o primeiro projeto com essa parceria, até porque a gente tinha recém-inaugurado… que eu saiba foi o primeiro. Depois outros vieram. Outros projetos e com outras unidades também da Fiocruz (Maciel, 15 fev. 2023).

A compreensão de que o Projeto Coleções teve papel essencial no processo de reconhecimento institucional e técnico-científico da COC não é uma conclusão automática para todos os entrevistados. Magali Romero Sá, primeiramente, avalia que o projeto não teria tido toda essa importância em perspectiva:

Eu não acho que seja esse projeto que tenha dado visibilidade pra COC, não, outros projetos, sim. Porque, quando eu entrei, ainda tinha muito problema.

… Eu entrava e conhecia todo mundo do IOC, era outro tipo de diálogo. Então eu não senti. Mas a COC estava com muita resistência ao trabalho e também pelo Museu da Vida. Colocar um museu dentro dessa instituição, justamente naquele período, era uma coisa horrorosa: ‘Vem público pra cá, isso aqui é uma instituição de pesquisa, tem laboratório, vai encher de gente! Vai percorrer as nossas alas!’. Era outra visão de mundo ... As instituições eram todas muito fechadas, e essa resistência foi muito grande para a COC, que foi avançando e, aos poucos, foi sendo quebrada (Sá, 25 jan. 2023).

A perspectiva diferenciada de Magali tem direta relação com o seu ângulo de visão específico. No momento de sua inserção no Projeto Coleções pela COC, Magali já tinha uma trajetória profissional na área biológica do IOC. Havia feito doutorado em história e filosofia das ciências e, diferentemente dos demais pesquisadores entrevistados para essa pesquisa, chegou ao projeto para desempenhar uma função de destaque, como coordenadora. A princípio, em sua memória, marcante foi a necessidade de sua experiência anterior no IOC para o desempenho daquela atribuição. Em seu raciocínio imediato, não fora essa inserção que teria iniciado uma mudança no curso de resistência do instituto à COC. Para ela, a resistência permanecera, já que, no IOC, sua sensação era a de que não a viam exatamente como uma historiadora da COC, mas como uma espécie de pesquisador híbrido, uma peça coringa naquele tabuleiro institucional que seguia igual.

Magali reconhece o trabalho desenvolvido no projeto para a maior integração da COC com as outras unidades da Fiocruz ao longo da entrevista, enquanto vai tecendo suas memórias e direcionando sua narrativa para as dificuldades ultrapassadas ao longo do tempo. A partir daí, confere reconhecimento aos dividendos que o Projeto Coleções deixou para a COC:

Hoje em dia não, todo mundo vai adorar fazer, mas, naquela época, tinha muita resistência. E essa resistência foi quebrada, quer dizer, na realidade, o projeto quebrou a resistência da Casa, sim. Eu estou minimizando a importância do projeto, depois de tantos anos ...

… eu acho que, no IOC, eles passaram a entender melhor a Casa, nesse sentido acho que, talvez a pergunta que tenha me feito antes, essa seria a resposta. Não que a Casa se tornou mais coisa, mas eles passaram a entender melhor a Casa. A respeitar mais a COC, nesse sentido, porque eles começaram a se ver dentro daquele processo (Sá, 25 jan. 2023).

A história do acervo Memória das Coleções Científicas do Instituto Oswaldo Cruz da Fiocruz aqui apresentada buscou compreender como certas escolhas, limitações e possibilidades estruturais podem ter contribuído para firmar essa memória e as próprias coleções científicas como patrimônio cultural das ciências e da saúde. A partir do texto do Projeto Papes (1993), do reuso das entrevistas resultantes do Projeto Coleções (1994-2001), do catálogo de entrevistas e do cotejo desses documentos com os depoimentos de 2023 feitos com pesquisadores da COC que trabalharam no projeto durante a sua execução, foi possível compreender que, de fato, o acervo de história oral de Memória das Coleções Científicas do Instituto Oswaldo Cruz da Fiocruz é parte constituinte de um processo de reconhecimento e consolidação da patrimonialização dessas coleções e de suas memórias engendrado pela Fiocruz. Processo esse que contribuiu também com importante ressignificação para pavimentar o caminho de reconhecimento institucional e técnico-científico trilhado pela COC.

O Projeto Coleções e os seus desdobramentos atribuíram maior projeção a parte das coleções científicas do IOC, conferiram mais visibilidade às memórias de seus curadores, ao próprio IOC, à COC e ao seu corpo de pesquisadores, além de, em última instância, à própria Fiocruz. É reflexo dessa engrenagem, por exemplo, a presente pesquisa. O conhecimento aqui gerado sobre a história da Memória das Coleções Científicas do Instituto Oswaldo Cruz da Fiocruz foi possível devido à existência e à disponibilização pela COC do acervo relacionado ao Projeto Coleções.

O processo de patrimonialização das coleções científicas do IOC, que abarcou a construção de suas memórias, traduz o real sentido que José Reginaldo Gonçalves (2003) confere à conceituação de patrimônio, que, nas mais diversas variações assumidas na contemporaneidade (econômica, financeira, cultural, arquitetônica, científica, entre outras), é usado não apenas para simbolizar, representar ou comunicar, mas também para agir. O trabalho de pesquisa apresentado preconiza exatamente essa noção de que o patrimônio não existe apenas para representar ideais, valores abstratos e para ser contemplado, “o patrimônio, de certo modo, constrói, forma as pessoas”. Um arquivo sobre a memória de coleções patrimonializadas traduz, reflete e age, de diferentes maneiras, no processo de formação e consolidação desse patrimônio, demarcando um domínio subjetivo, criando uma identidade para as coleções, seus colecionadores, acervos e entidades custodiadoras (Gonçalves, 2003, p.22-27).

O caso de patrimonialização aqui estudado descortina contingências que conformaram a constituição do acervo de entrevistas intitulado Memória das Coleções Científicas do Instituto Oswaldo Cruz da Fiocruz, que hoje está depositado no Departamento de Arquivo e Documentação da COC/Fiocruz. O reuso desse acervo de história oral aconteceu a partir do questionamento sobre, em que medida, a narrativa por ele lançada refletia e era condicionada pelo contexto histórico vivenciado pelos profissionais e pela própria Fiocruz no período de sua produção. Por meio de novas perguntas sobre aquele acervo de entrevistas, tornou-se possível revelar algumas condições institucionais do momento de sua criação que estavam nele refletidas e que foram por ele ativadas. Uma vez mais, constatou-se que o estudo da história sobre a memória dos arquivos e das coleções é um caminho de investigação que confere historicidade a esses artefatos, contribuindo para a compreensão de subjetividades aparentemente imperceptíveis, mas que presidem a constituição, a organização de acervos e os significados atribuídos por meio de seus processos de patrimonialização e manutenção.

## NOTAS

1 Coleção Entomológica; Coleção de Culturas de Fungos; Coleção de Fungos Potencialmente Produtores de Micotoxinas e de Interesse em Saúde Coletiva; Coleção Cultura de Bactérias; Coleção de Culturas do Gênero Bacillus e Gêneros Correlatos; Coleção Febre Amarela; Coleção Helmintológica; Coleção de Leishmania; Coleção Malacológica. Disponível em: http://www.fiocruz.br/ioc/cgi/cgilua.exe/sys/start.htm?sid=7. Acesso em: 31 jul. 2023.

2 As coleções referenciadas são: Coleção de Bacillus; Coleção de Culturas do Instituto Nacional de Controle de Qualidade em Saúde (INCQS); Coleção Entomológica do Instituto Oswaldo Cruz; Coleção Helmintológica; Coleção de Leishmania; Coleção Micológica; Coleção de Isolados de *Trypanosoma cruzi*. Em seguida, o texto também menciona: “A Coleção de Tripanossomatídeos, a Coleção de Febre Amarela e a Coleção Malacológica, entre outros conjuntos tipológicos mais específicos, como a Coleção Coprológica, a de cracas, a de lâminas de lepra, a de isolados de *Mycobacterium tuberculosis*, a de isolados de vírus de *hepatitis*, pólio, rotavírus, vírus respiratório, bancos de células etc.” (Degrave, Albuquerque, 1993, p.6-6.1).

3 São os seguintes conjuntos: *Leishmania*, Gênero *Bacillus*, Genes de *Trypanossona cruzi* e Tripanossomatídeos (doença de Chagas), os dois últimos relacionados ao INCQS (Degrave, Albuquerque, 1993, p.6.3).

4 Os oitos pesquisadores do IOC com os direitos políticos cassados em 1970 foram: Haity Moussatché; Herman Lent; Moacyr Vaz de Andrade; Augusto Cid de Mello Perissé; Hugo de Souza Lopes; Sebastião José de Oliveira; Fernando Braga Ubatuba; Tito Cavalcanti. Em seguida, os mesmos oito foram compulsoriamente aposentados, juntamente com outros dois: Domingos Arthur Machado Filho e Masao Goto (Hamilton, 1989).

5 Entre 1995 e 1999, Flávio Edler se doutorou em saúde coletiva na Universidade do Estado do Rio de Janeiro. Sobre o tema, produziu trabalhos importantes, entre os quais sua tese (Edler, 1999) e alguns artigos científicos publicados nesta revista (Edler, 1995, 1996, 2002, 2010).
